# Supplementary material for: Evaluation of the Emulsification Properties of Marine-Derived Rhamnolipids for Encapsulation: A Comparison with Commercial Surfactants
Source: Biomolecules. 2025 Oct 14;15(10):1451. doi: 10.3390/biom15101451 (PMC12564215; doi:10.3390/biom15101451)
Supplement: Supplementary file 1 [file biomolecules-15-01451-s001.zip › biomolecules-3837067-supplementary.pdf]

Table of Contents

S1. Mass spectrometry of M15CE and M15RL mixtures

Table S1. RLs congeners differences from the control (pH 7, RT), in terms of relative abundance, in the samples treated at 90 °C, pH 3, and pH 12.

Figure S1: Surface tension vs. concentration plots (surface adsorption isotherms) for the tested surfactants. Panels show: A) M15CE, B) M15RL, C) SDS, and D) SE. The CMC of each surfactant was determined from the intersection of the descending slope with the plateau region, as indicated in the plots. Measurements were performed in triplicate, and error bars represent the standard deviation.

Figure S2: Schematic representation of an oil-in-water emulsion. The left tube illustrates a homogeneous emulsion, while the right tube shows phase separation into two layers: an emulsion layer and a cream layer on the top, enriched in oil droplets that have migrated upward due to gravitational separation.

Figure S3. Visual appearance of emulsions prepared with different biosurfactants (M15CE, SDS and SE at a concentration of 0.2 mg/ml). at varying oil-to-water ratios. The images illustrate the extent of phase separation as a function of formulation. **A)** oil-water ratio 1:1, **B)** oil-water ratio 1:2,5, **C)** oil-water ratio 1:5 and **D)** oil-water ratio 1:10.

Figure S4. Droplet size distributions of emulsions stabilized with M15CE, SDS, and SE at different oil-to-water ratios: **A)** 1:1, **B)** 1:2.5, **C)** 1:5, and **D)** 1:10. Surfactant concentration tested was 0.2 mg/ml.

Figure S5: Optical microscopy images of emulsion droplets at an oil-to-water ratio of 1:10 and a surfactant concentration of 0.2 mg/mL. Images were acquired with an optical microscope (Optotech, Germany), at 100× magnification , with scale bars of 1 µm and 10 µm. Panels show emulsions stabilized with: A) M15CE, B) M15RL, C) SDS, and D) SE.

Figure S6. Evolution of emulsion droplet size distributions over time at room temperature and at 40°C (dotted line). A) droplet size distributions after emulsification step (t0), B) droplet size distributions after 24h, and C) droplet size distributions after 1 week.

S1. Mass spectrometry of M15CE and M15RL mixtures

The RL mixtures were dissolved in MeOH at 1 mg/mL and analyzed through Liquid Chromatography—High-Resolution Tandem Mass Spectrometry (LC-HRMS^2). The chemical profiling was acquired with a Thermo Scientific Q Exactive Focus Orbitrap mass spectrometer coupled to a Thermo Scientific Ultimate 3000 HPLC system equipped with a Hypersil C18 (RPC18) column (100 x 4.6 mm, 3 µm) (Thermo Fisher Scientific, Waltham, MA, USA). The RP18 was eluted at 25 °C at 200 µL/min with H_2O (A) and CH_3CN (B), both supplemented with 0.1% formic acid. The gradient was set as follows: 25-60% B for 5 min, 60-100% B for 30 min, and 100% B for 20 min. MS spectra were acquired in negative ion mode. HESI source parameters were set as follows: sheath gas flow rate to 45 units N_2, auxiliary gas flow rate to 10 units N_2, spray voltage to 3.2 kV, capillary temperature to 285 °C, and auxiliary gas heater temperature to 150 °C. The full MS were acquired in the scan range of 150–1000 m/z with a resolution of 70,000 and an AGC target of 1e^6. MS^2 spectra were acquired in the data-dependent analysis mode at a resolution of 70,000 and an AGC target of 5e^4, setting three MS^2 events after each full MS scan. HRMS^2 scans were obtained with HCD fragmentation, using an isolation width of 2.0 m/z, and normalized collision energies of 20 and 30 units.

Table S*2*. RLs congeners differences from the control (pH 7, RT), in terms of relative abundance, in the samples treated at 90 °C, pH 3, and pH 12.

Rhamnolipids[1]	Base Peak	Δ 90 °C [2]	SEM	Δ pH 3 [2]	SEM	Δ pH 12 [2]	SEM
			M15CE				
Rha-C12:1	359.2075	0.1%	3.72E-04	0.5%	4.77E-04	-0.1%	1.24E-03
Rha-C12	361.2231	0.2%	7.92E-04	1.2%	2.11E-03	-0.6%	5.47E-03
Rha-C8-C10	475.2907	1.1%	1.35E-02	0.6%	2.68E-02	-4.5%	1.72E-02
Rha-C10-C10	503.3223	1.6%	1.78E-02	-0.6%	2.56E-02	-2.6%	2.23E-03
Rha-C12:1-C10	529.3380	-1.6%	2.85E-02	-0.6%	4.10E-02	3.5%	2.79E-02
Rha-C12-C10	531.3538	-2.5%	2.99E-02	-1.0%	4.21E-02	2.1%	1.75E-02
Rha-C12:1-C12:1	555.3538	0.1%	3.16E-03	-0.1%	2.83E-03	0.7%	1.63E-03
Rha-C14:1-C10	557.3694	-0.2%	1.48E-03	-0.1%	1.95E-04	0.9%	3.41E-03
Rha-C12-C12	559.3850	-0.3%	4.26E-03	-0.5%	3.74E-03	0.4%	2.60E-04
			M15RL				
Rha-C8-C10	475.2907	-1.0%	2.84E-03	0.2%	1.94E-02	-5.8%	8.76E-05
Rha-C10-C10	503.3223	-1.3%	2.10E-03	-2.1%	1.40E-02	-4.9%	1.97E-02
Rha-C11-C10	517.3380	-0.6%	1.57E-04	-0.8%	3.65E-03	-0.6%	4.70E-03
Rha-C12:1-C10	529.3380	2.5%	2.70E-03	2.6%	2.82E-02	5.9%	7.65E-03
Rha-C12-C10	531.3538	2.2%	3.59E-04	2.3%	1.88E-02	4.6%	9.47E-03
Rha-C14:1-C10	557.3694	-0.2%	8.38E-04	-0.1%	9.77E-04	0.6%	3.18E-03
Rha-C12-C12:1	557.3694	-0.2%	2.59E-03	-0.2%	2.74E-04	0.6%	8.37E-04
Rha-C12-C12	559.3850	1.2%	5.20E-03	0.2%	1.54E-03	1.4%	1.31E-02
Rha-C14-C10	559.3850	-1.0%	5.21E-03	-0.1%	6.79E-04	-0.7%	7.94E-03

[1] *Rha = rhamnose. Fatty acyl chains are indicated as Cn:x, where n is the number of carbon atoms, and x is the number of double bonds.* [2] *Relative abundance variation from the control.* Treshold 0.5%. *SEM= Standard error median, expressed in scientific notation.*

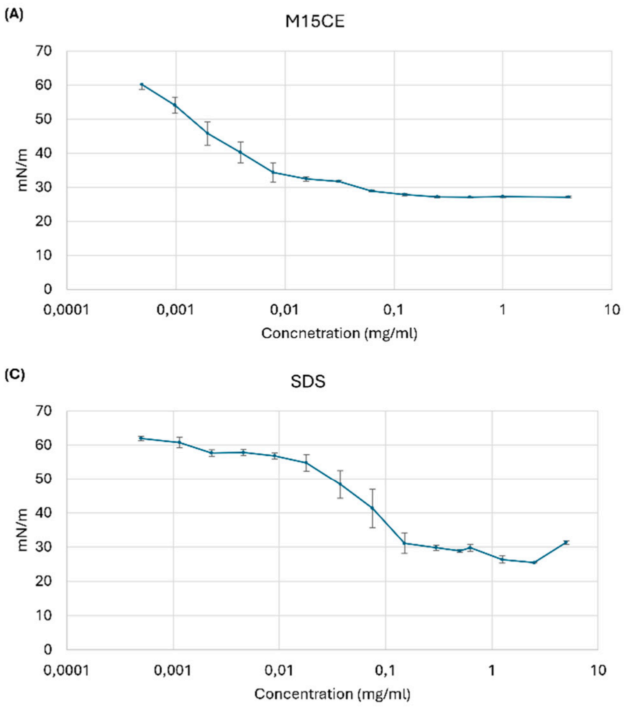

Figure S1: Surface tension vs. concentration plots (surface adsorption isotherms) for the tested surfactants. Panels show: **(A)** M15CE, **(B)** M15RL, **(C)** SDS, and **(D)** SE. The CMC of each surfactant was determined from the intersection of the descending slope with the plateau region, as indicated in the plots. Measurements were performed in triplicate, and error bars represent the standard deviation.

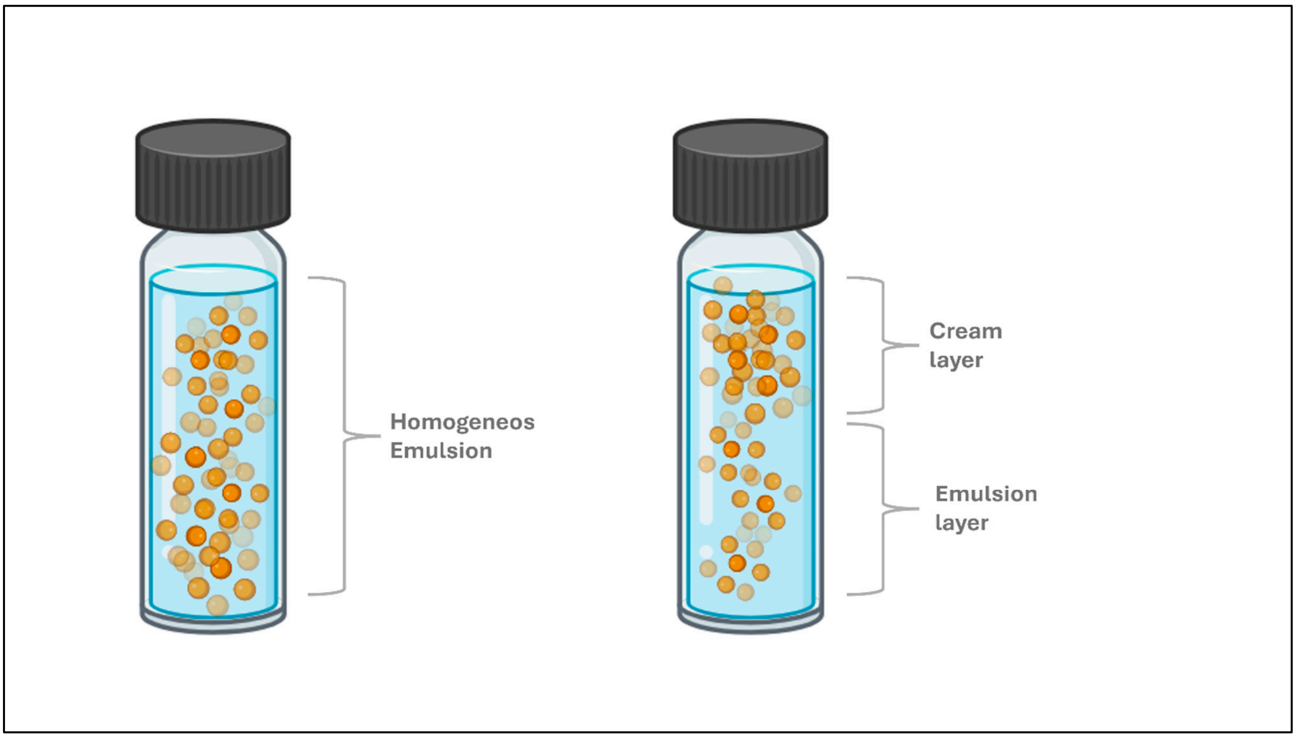

Figure S2: Schematic representation of an oil-in-water emulsion. The left tube illustrates a homogeneous emulsion, while the right tube shows phase separation into two layers: an emulsion layer and a cream layer on the top, enriched in oil droplets that have migrated upward due to gravitational separation.

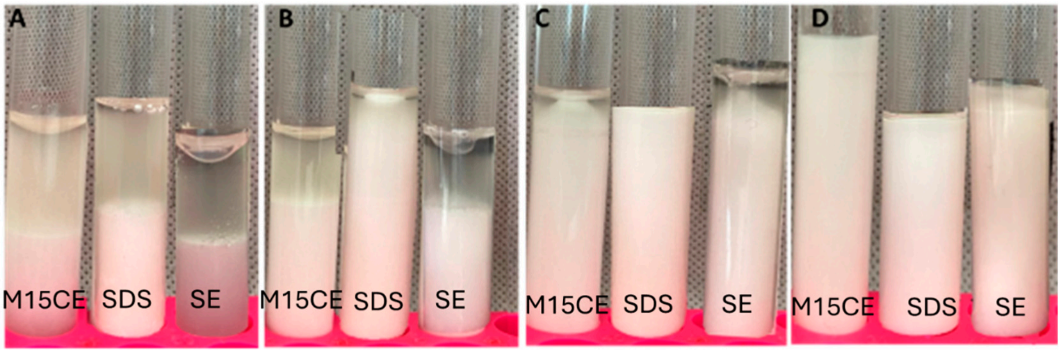

Figure S3. Visual appearance of emulsions prepared with different biosurfactants (M15CE, SDS and SE at a concentration of 0.2 mg/ml). at varying oil-to-water ratios. The images illustrate the extent of phase separation as a function of formulation. **(A)** oil-water ratio 1:1, **(B)** oil-water ratio 1:2,5, **(C)** oil-water ratio 1:5 and **(D)** oil-water ratio 1:10.

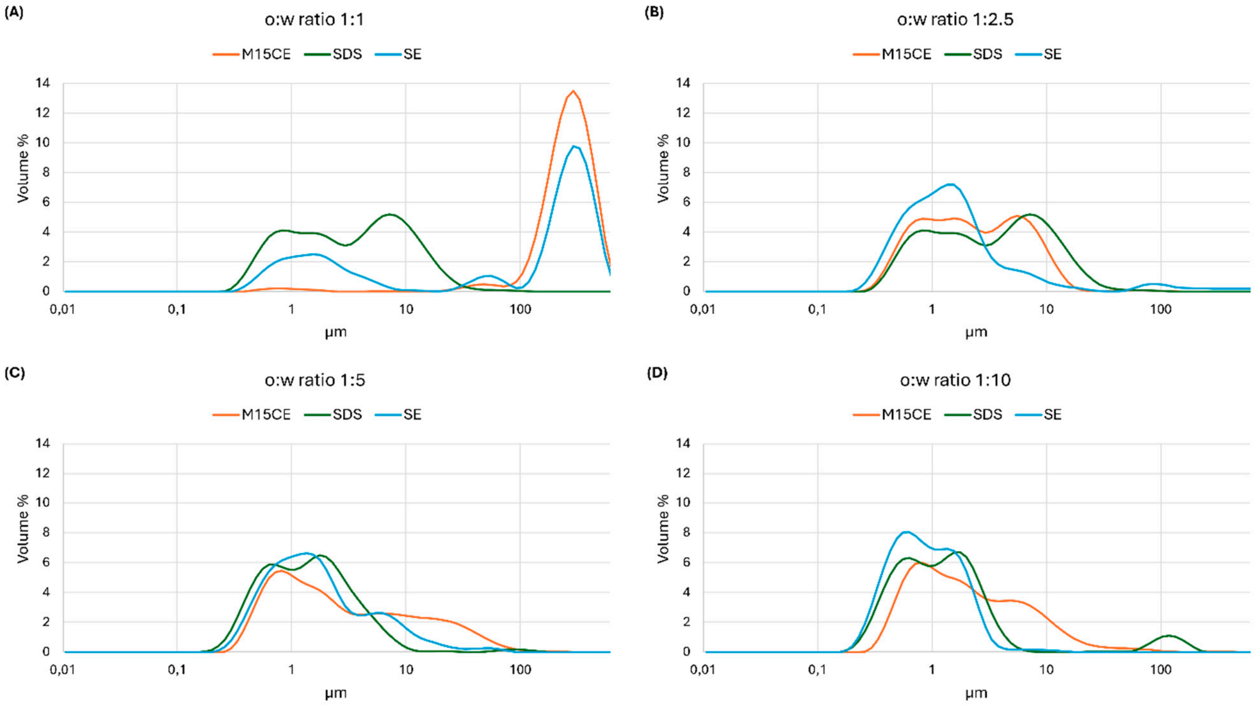

Figure S4. Droplet size distributions of emulsions stabilized with M15CE, SDS, and SE at different oil-to-water ratios: **(A)** 1:1, **(B)** 1:2.5, **(C)** 1:5, and **(D)** 1:10. Surfactant concentration was 0.2 mg/ml.

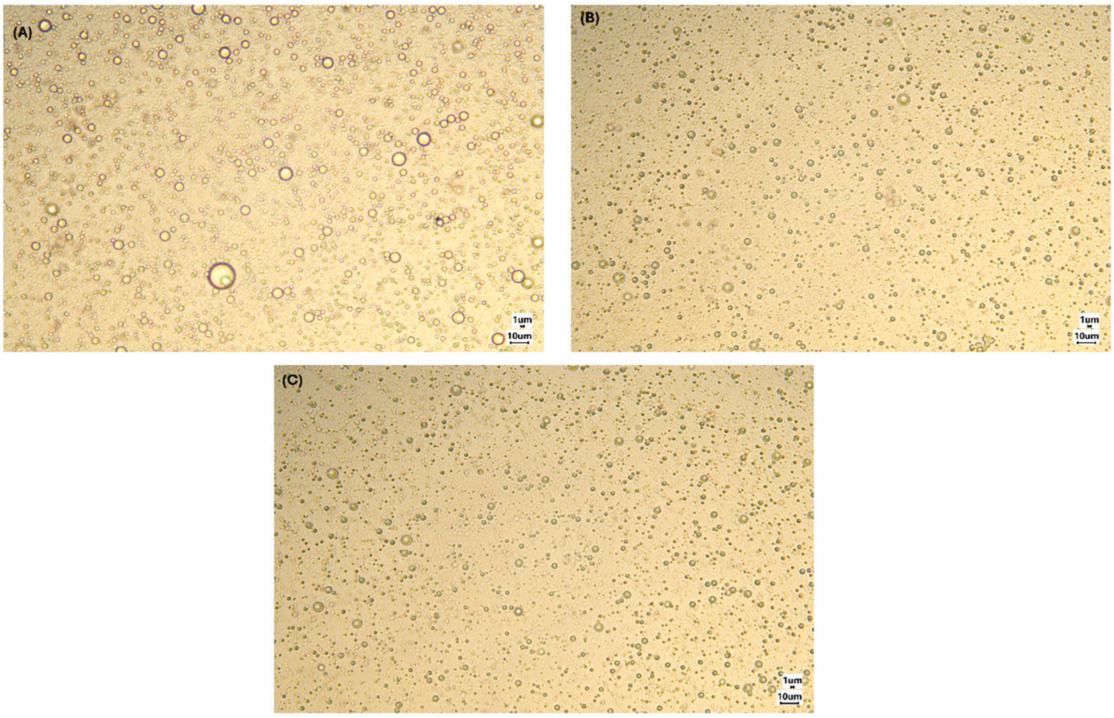

Figure S5: Optical microscopy images of emulsion droplets at an oil-to-water ratio of 1:10 and a surfactant concentration of 0.2 mg/mL. Images were acquired through an optical microscope (Optotech, Germany) at 100× magnification, with scale bars of 1 µm and 10 µm. Panels show emulsions stabilized with: **(A)** M15CE, **(B)** SDS, and **(C)** SE.

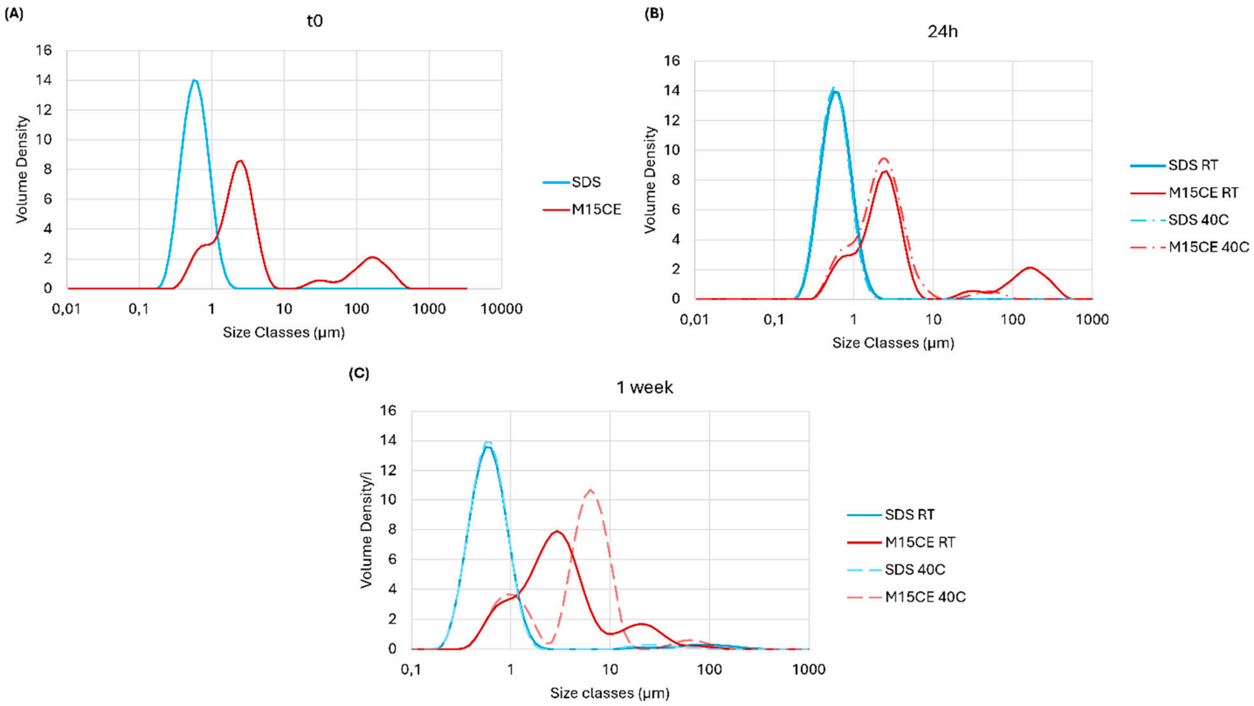

Figure S6: Evolution of emulsion droplet size distributions over time at room temperature (RT) and at 40°C (dotted line). **(A)** droplet size distributions after emulsification step (t0), **(B)** droplet size distributions after 24h, and **(C)** droplet size distributions after 1 week.
